# Supplementary material for: Complexities in the role of acetylation dynamics in modifying inducible gene activation parameters
Source: Nucleic Acids Res. 2021 Dec 1;49(22):12744–56. doi: 10.1093/nar/gkab1176 (PMC8682737; doi:10.1093/nar/gkab1176)
Supplement: gkab1176_Supplemental_Files [file gkab1176_supplemental_files.zip › Extra figure captions.docx]

**NAR-01786-J-2021.R1: Additional Legends**

**Supplementary Table S1.** PCR primers used in this study for CRISPR-mediated cell line construction, RT-qPCR and ChIP-qPCR.

**Supplementary Table S2**. List of EGF inducible genes identified from RNAseq analysis.

**Movie 1**: Single cell luciferase reporter expression for the tagged EGR2 locus. MCF10A-EGR2-Luc cells were serum starved for 48 hours and then treated with EGF and imaged over a 5 hour time period.

**Movie 2**: Single cell luciferase reporter expression for the tagged DUSP1 locus. MCF10A-DUSP1-Luc cells were serum starved for 48 hours and then treated with EGF and imaged over a 5 hour time period.
